# Characterization of FA1654: A putative DPS protein in *Filifactor alocis*

**DOI:** 10.1111/omi.12398

**Published:** 2022-12-19

**Authors:** Malissa Mangar, Arunima Mishra, Zhengrong Yang, Champion Deivanayagam, Hansel M. Fletcher

**Affiliations:** ^1^ Division of Microbiology & Molecular Genetics School of Medicine Loma Linda University Loma Linda California USA; ^2^ Department of Biochemistry and Molecular Genetics University of Alabama Birmingham Alabama USA

**Keywords:** DPS protein, Fenton reaction, *Filifactor alocis*, oxidative stress, periodontal disease

## Abstract

The survival/adaptation of *Filifactor alocis*, a fastidious Gram‐positive asaccharolytic anaerobe, to the inflammatory environment of the periodontal pocket requires an ability to overcome oxidative stress. Moreover, its pathogenic characteristics are highlighted by its capacity to survive in the oxidative‐stress microenvironment of the periodontal pocket and a likely ability to modulate the microbial community dynamics. There is still a significant gap in our understanding of its mechanism of oxidative stress resistance and its impact on the virulence and pathogenicity of the microbial biofilm. Coinfection of epithelial cells with *F. alocis* and *Porphyromonas gingivalis* resulted in the upregulation of several genes, including *HMPREF0389_01654 (FA1654)*. Bioinformatics analysis indicates that FA1654 has a “di‐iron binding domain” and could function as a DNA starvation and stationary phase protection (DPS) protein. We have further characterized the FA1654 protein to determine its role in oxidative stress resistance in *F. alocis*. In the presence of hydrogen peroxide‐induced oxidative stress, there was an ∼1.3 fold upregulation of the *FA1654* gene in *F. alocis*. Incubation of the purified FA1654 protein with DNA in the presence of hydrogen peroxide and iron resulted in the protection of the DNA from Fenton‐mediated degradation. Circular dichroism and differential scanning fluorimetry studies have documented the intrinsic ability of rFA1654 protein to bind iron; however, the rFA1654 protein is missing the intrinsic ability to reduce hydrogen peroxide. Collectively, the data may suggest that FA1654 in *F. alocis* is involved in oxidative stress resistance via an ability to protect against Fenton‐mediated oxidative stress‐induced damage.

**List of abbreviations**
Ahpc/Ahpf: Alkyl hydroperoxide reductase
Asp: Aspartic acid
ATCC: American type culture collection
Bcp: Bacterioferritincomigratory protein
BHI: Brain heart infusion
CD: Circular dichroism
CO_2_
: Carbon dioxide
DNA: Deoxyribonucleic acid
DPS: DNA starvation and stationary phase protection protein
DSF: Differential scanning fluorimetry
FA: *Filifactor alocis*
Fe: Iron
FEOOH: Iron oxide hydroxide
Fe_2_SO_4_
: Iron(II) sulfate
H_2_
: Hydrogen
H_2_O_2_
: Hydrogen peroxide
IPTG: Isopropyl β‐D‐1‐thiogalactopyranoside
I‐TASSER: Iterative Threading Assembly Refinement
kDA: Kilodalton
LB: Luria‐Bertani
LPS: Lipopolysaccharide
N_2_
: Nitrogen
NaCl: Sodium chloride
OD: Optical density
PCR: Polymerase chain reaction
qPCR: Real time polymerase chain reaction
RNA: Ribonucleic acid
ROS: Reactive oxygen species
RT‐PCR: Reverse transcriptase polymerase chain reaction
SDS‐PAGE: Sodium Dodecyl Sulfate‐Polyacrylamide Gel Electrophoresis
SOR: Superoxide reductase
T_m_
: Thermal unfolding temperature
TMB:  3,5,3′5′‐tetramethylbenzidine
Trp: Tryptophan

## 1 INTRODUCTION

Bacteria in the periodontal pocket are consistently exposed to oxidative stress (Ortiz de Orué Lucana et al., 2012). Oxidative stress can be described as a disruption in the balance of reactive oxygen species (ROS), such as superoxide (O_2_^−^), hydrogen peroxide (H_2_O_2_), hydroxyl radical (OH•), and nitric oxide (NO), in the environment and the ability of biological systems to cope with this dysregulation (Aja, Mishra, et al., 2021; Ortiz de Orué Lucana et al., 2012; Sczepanik et al., 2020). ROS are generated as a result of immune responses, such as phagocytosis which triggers the activation of NADPH oxidase (DeLeo et al., 1999), to pathogenic infection and as a result of aerobic metabolism (Aja, Mishra, et al., 2021; Sczepanik et al., 2020). Oxidative stress can be detrimental to the survival of organisms and can impede the integrity of lipids, proteins, DNA and RNA, and other membrane structures (Henry et al., 2012; Ortiz de Orué Lucana et al., 2012). Bacterial species, however, have evolved and developed sophisticated regulatory, repair, and protective mechanisms to enable the detection of oxidative stress and produce an effective and efficient response to mediate any damage that may ensue. One such mechanism is DNA starvation and stationary phase protection (DPS) protein and DPS‐like proteins (Bellapadrona et al., 2010; Karas et al., 2015; Pulliainen et al., 2005; Tseng et al., 2019).

DPS and DPS‐like proteins (DPR) are members of the Ferritin super family and protect DNA from oxidative stress damage (Chiancone & Ceci, 2010; Pulliainen et al., 2005). DPS and DPS‐like proteins are dodecamers, with a four helix structure (Bellapadrona et al., 2010). Protection against oxidative stress‐induced DNA damage by these proteins occurs via two mechanisms. DNA binding provides physical protection for DNA from damaging agents such as ROS. Chemical protection is provided via the proteins’ ferroxidase center, which may lead to the oxidation of iron from its Fe^2+^ to Fe^3+^ state, using hydrogen peroxide as the oxidizing agent.

*Filifactor alocis* is a gram‐positive, rod shaped obligate anaerobe. Upon its initial isolation from the gingival sulcus in 1985, *F. alocis* was classified as *Fusobacterium* species, to be later reclassified as *Filifactor* sp. in 1999 (Aja, Mangar, et al., 2021; Cato et al., 1985; Moffatt et al., 2011). *F. alocis* is present to a higher extent than the “Red complex” species; *Porphyromonas gingivalis*; the keystone pathogen, *Treponema denticola*, and *Tannerella forsythia* (Aruni, Chioma, et al., 2014; Chen et al., 2015; Hajishengallis & Lamont, 2012); in patients with periodontal disease, whilst being relatively undetected in healthy patients. This implies that it plays a role in the onset of periodontal disease. Periodontal disease is an inflammatory disease affecting the integrity of the supporting structures of the teeth and is characterized by the destruction of periodontal tissue and loss of alveolar bone (Aja, Mangar, et al., 2021; Moffatt et al., 2011). Periodontal disease has been linked to systemic diseases such as diabetes, obesity, pre‐termed labor, and Alzheimer's (Bascones‐Martinez et al., 2014; Kuo et al., 2008).

*F. alocis* has been shown to increase the resistance of oral pathogens such as *P. gingivalis* to oxidative stress. A previously conducted study by our lab demonstrated an increased resistance in excess of fourfold in *P. gingivalis* to hydrogen peroxide‐induced oxidative stress in coculture with *F. alocis* (Aja, Mishra, et al., 2021; Aruni et al., 2011, 2012), along with increased invasion and adhesion abilities of epithelial cells, TGIKs and NOKs (Aja, Mishra, et al., 2021; Aruni, Zhang, et al., 2014; Mishra et al., 2020). Recently a superoxide reductase (SOR) along with a multifunctional protein with peroxidase and disulfide oxo‐reductase activity, FA519, have been determined to play a role in shielding *F. alocis* from oxidative stress (Aja, Mishra, et al., 2021; Mishra et al., 2020).

Despite the progress understanding the virulence mechanism of *F. alocis*, a gap in our understanding the role of other genes in the oxidative stress resistance mechanism of *F. alocis* still exists. In a coinfection study of epithelial cells with *F. alocis* with *P. gingivalis*, several genes were upregulated, one of which was *FA1654*, this gene encodes for the hypothetical protein FA1654 (Aruni et al., 2012).

Bioinformatics analysis of FA1654, a hypothetical protein with DPS‐like function in *F. alocis*, has shown that the protein has a putative di‐iron binding domain and is approximately 16 kDa in size. In this study, we characterize FA1654 to determine its role in oxidative stress resistance and in the virulence mechanism of the pathogen.

## 2 MATERIALS AND METHODS

### 2.1 Bacterial strains and growth conditions

The bacterial strains and plasmids used in this study are listed in Table 1. *F. alocis* strain ATCC 35896 was cultured in Brain Heart Infusion broth supplemented with 0.5 µg/ml vitamin K, 5 µg/ml hemin, 0.1% cysteine, and 100 µm arginine. *F. alocis* was grown anaerobically at 37°C and maintained in an anaerobic chamber (Coy Manufacturing in 10% H_2_, 10% CO_2_, and 80% N_2_). TOP10 and BL21 *Escherichia coli* strains were cultured in Luria‐Bertani (LB) broth supplemented with 100 µg/ml ampicillin at 37°C. Growth rates of bacterial cultures were determined by measuring the optical density at 600 nm (OD_600_).

**TABLE 1:** Bacterial strains and plasmids used in this study

Strains	Source
*Filifactor alocis* 35896	ATCC
*Escherichia coli* TOP10	Life Technology
*E. coli* BL21	Life Technology
Plasmid	Thermo Fisher Scientific
pET 102 D‐TOPO	

### 2.2 qPCR analysis

Primers generated for the gene of interest are listed in Table 2. The amplification of the genes of interest was performed with the SYBR green kit (Qiagen), and the signal detection for real‐time fluorescence was done via the Cepheid Smart Cycler Real Time PCR device. The cycles are as follows: 95°C for 15 min, 40 cycles of 94°C for 15 s, 54°C for 30 s, and 72°C for 30 s. Measurements were done in triplicate, and 16S rRNA was utilized as an internal control (Dou et al., 2014).

**TABLE 2:** Primers used in this study

Use and name of primer	Sequence (5′–3′)
Overexpression of FA1654	
FA1654‐pET 102‐F1	CACCATGATGAACTTAAAAAAAGAATTT
FA1654‐pET 102‐R1	TTTCACGCTTGAGTTAATGAAC
qPCR analysis	
F1	CAAAGAGGTGGATCCAAGAGAA
R1	CTTCTGTCCAACCCAATCCA

### 2.3 Bioinformatics analysis

The nucleotide sequence of the *FA1654* (from *F. alocis* ATCC 35896) and BLAST analysis was retrieved from the NCBI database (Mishra et al., 2020). The structural predictions of the protein were done using SWISS‐MODEL (https://swissmodel.expasy.org/) (Mishra et al., 2020) and the I‐TASSER database (https://zhanglab.dcmb.med.umich.edu/I‐TASSER/).

### 2.4 Cloning, expression, and purification of rFA1654

In order to characterize FA1654, the *FA1654* gene was amplified from *F. alocis* wild‐type genomic DNA, using PCR. The amplified gene product was then cloned into pET 102/D‐TOPO vector using the TOPO expression kit from Life Technologies. The pET 102/D‐TOPO vector with the *FA1654* gene is then transformed into *E. coli* BL‐21 cell. BL‐21 cells were cultured in LB broth supplemented with 100 mg/ml ampicillin and grown overnight in a 37°C shaker. At an OD of 0.6, 0.5 mM IPTG was used to induce the expression of the protein and the culture grown for an additional 4 h. The culture was then centrifuged. The pellet was collected and resuspended in lysis buffer (150 mM NaCl, 50 mM Tris buffer, pH 7.5, 10 mM imidazole) and mechanically lysed using French press. The “lysate” was then transferred to a nickel column to allow for the elution of the protein. The protein is then collected and dialyzed in EQ buffer with 10% glycerol at 4°C 16 h. Dialysis was repeated once more (Mishra et al., 2020).

### 2.5 DNA protection assay

Purified FA1654 (2–8 µm final concentration) was incubated with DNAJ, FA1654, and RNA polymerase B (DNA template) in Tris buffer (20 mM, pH 7.8), FeSO_4_ (50 µM), and deionized water for 5 min at room temperature. H_2_O_2_ with a concentration of 88–264 mM, as necessary, was then added to the reaction mix and further incubated for 30 min at room temperature. A 10× loading dye was then added to the reaction and subsequently resolved via agarose gel electrophoresis using a 1% agarose gel stained with ethidium bromide (Karas et al., 2015).

### 2.6 DNA binding assay

Purified FA1654 (2–8 µm final concentration) was incubated with pUC19 plasmid DNA in Tris buffer (20 mM, pH 7.8), FeSO_4_ (50 µM), and deionized water for 5 min at room temperature. H_2_O_2_ with a concentration of 88–264 mM, as necessary, was then added to the reaction mix and further incubated for 30 min at room temperature. A 10× loading dye was then added to the reaction and subsequently resolved via agarose gel electrophoresis using a 1% agarose gel stained with ethidium bromide (Karas et al., 2015).

### 2.7 H_2_O_2_ detection using the 3,5,3′5′‐tetramethylbenzidine (TMB) assay

A typical colorimetric analysis was performed as follows: First, fresh 10 mM stocks of H_2_O_2_ and FeSO_4_ were prepared. The FA1654 samples (0.2 mM) were then incubated with 2 mM FeSO_4_, 1 mM H_2_O_2_, and 0.7 mM TMB (3,5,3′5′‐tetramethylbenzidine) in 0.1 M sodium phosphate buffer (pH 6.0) for 15 min at room temperature. The reaction was then terminated by the addition of 50 µl 0.5 M sulfuric acid (Rhee et al., 2010). Finally, the reaction mixture was used for absorption spectroscopy measurement at 450 nm via a SpectraMax® i3xPlate reader with the SoftMax® Pro 6 (version6.5.1) software.

### 2.8 Circular dichroism analysis

Circular dichroism (CD) spectra were recorded in an OLIS CD spectrophotometer (OLIS, Athens, GA). Quartz cuvettes with a path‐length of 0.02 cm were used. The protein concentration was 0.8 mg/ml. The CD cell holder was kept at 15°C using an external water bath. Two consecutive CD spectra were recorded from 260 to 195 nm for each sample with an integration time of 8 s/nm. Buffer baselines were recorded at the same temperature and subtracted from the protein spectra. The two protein spectra were averaged and concentration‐normalized to obtain molar ellipticity. The secondary structural content was determined using the CDNN software (CDNN: CD Spectra Deconvolution, Version 2.1, Universität Regensburg—Böhm, 1997).

### 2.9 Differential scanning fluorimetry

Thermal unfolding temperature (*T*_m_) of the protein with or without iron sulfate was determined using the Prometheus NT.48 NanoDSF instrument (NanoTemper Technologies, LLC, South San Francisco, CA) with 48 capillary chambers. Each sample was excited at 290 nm, and emission was detected simultaneously at 330 and 350 nm. The samples were heated from 15 to 95°C using a constant heating rate of 3°C/min. The first derivative of the fluorescence signal at 350 nm relative to 330 nm (F350/F330) versus temperature produced a bell‐shaped thermal unfolding peak. The midpoint of the peak corresponded to the *T*_m_. The *T*_m_ of each sample was automatically determined by the built‐in analysis software and tabulated in an Excel output file.

## 3 RESULTS

### 3.1 *F. alocis HMPREF0389_01654 (FA1654)* encodes for a hypothetical protein with an iron binding domain

**FIGURE 1:**
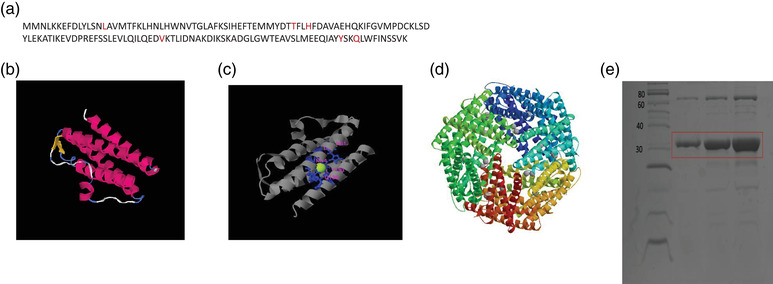
In silico analysis of FA1654, FA1654 is a putative DPS protein: (a) amino acid sequence of FA1654; (b) a structural prediction of FA1654 based on I‐TASSER modeling; (c) a structural prediction of putative iron binding sites of FA1654; (d) dodecameric structure of FA1654 based on SWISS‐MODEL and (e) SDS polyacrylamide gel electrophoresis (SDS–PAGE) gel showing rFA1654 with molecular weight ∼33.7 kDa.

The genome of *F. alocis* carries the *FA* *1654* gene, which is 437 base pairs in length and encodes for a 145 amino acid hypothetical protein that is approximately 16.7 kDa in size (https://www.ncbi.nlm.nih.gov/protein/504028302) (Figure 1a). Protein modeling via the I‐TASSER software indicates that this protein has a predictive iron binding site at amino acid positions 15, 48, 51, 100, 133, and 136 (Figure 1b and 1c) SWISS‐MODEL indicates the protein has a dodecameric tertiary structure (Figure 1d). There were no predicted DNA binding domains in FA1654. The amino acid sequence blast showed that FA1654 is annotated as a putative DPS protein that has an identity of 62.24%, 58.04%, and 47.92% to *Erysipelotrichaceae bacterium*, *Treponema pedis*, and *Fusobacterium* spp., respectively. The *E. coli* purified recombinant FA1654 was resolved on an SDS polyacrylamide gel electrophoresis gel as an ∼33.7 kDa thioredoxin‐His tag fusion protein (Figure 1e).

### 3.2 FA1654 is upregulated under hydrogen peroxide‐induced stress

**FIGURE 2:**
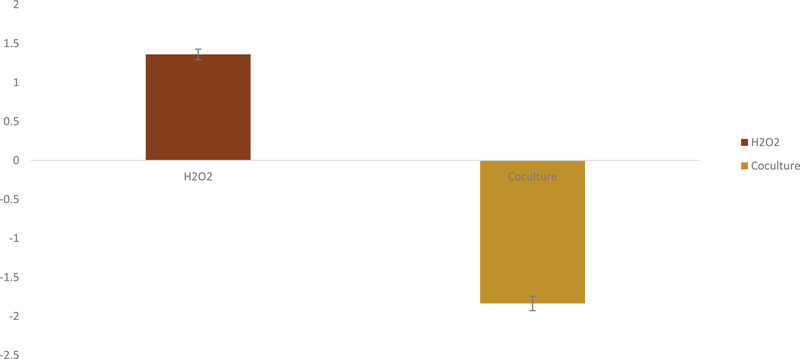
*FA1654* is upregulated when *F. alocis is* in the presence of H_2_O_2_‐induced oxidative stress. RT‐qPCR was conducted on a monoculture of *F. alocis* incubated with 0.25 mM of H_2_O_2_ for 15 min. *F. alocis* in coculture with *P gingivalis* was incubated with H_2_O_2_ for 15 min.

In the presence of epithelial cells, there was an upregulation of the *FA1654* gene in *F. alocis* (Aruni et al., 2012). RT‐PCR was used to analyze the expression of the *FA1654* gene in *F. alocis* under both anaerobic and oxidative stress conditions. As shown in Figure 2, the expression of the *FA1654* gene was induced by ∼1.3 fold when *F. alocis* was exposed to 0.25 mM of hydrogen peroxide for 30 min. However, under coculture conditions with *P. gingivalis*, the *FA1654* gene was downregulated ∼1.8 fold. It is likely that this could be the result of a compensatory oxidative stress mechanism that is activated under coculture conditions (Aja, Mishra, et al., 2021).

### 3.3 FA1654 protects DNA from Fenton reaction‐mediated damage

**FIGURE 3:**
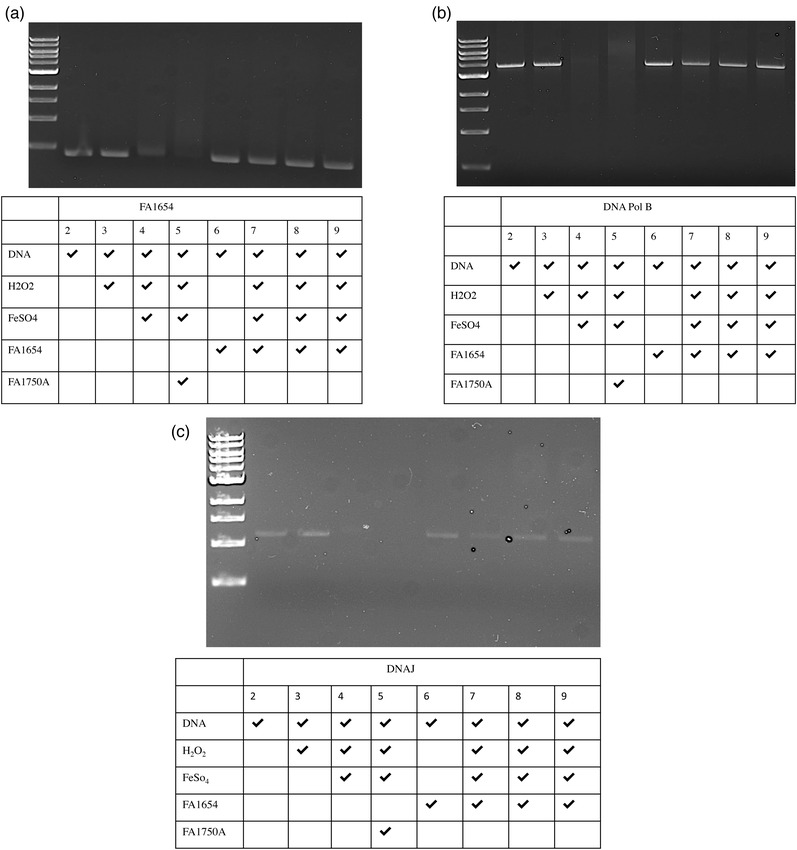
rFA1654 protects DNA from oxidative damage in vitro. The 1% agarose gels shown represent DNA protection assays. (a–c) Lane 1, TriDye 1 kb Ladder; lane 2, DNA alone (positive control); lane 3 DNA plus H_2_O_2_; lane 4, DNA plus Fenton reaction (Fe^2+^ and H_2_O_2_); lane 5, DNA plus Fenton reaction plus FA1750 (subunit a) (negative control); lane 6, DNA plus 2 µM rFA1654; lanes 7–9, DNA plus full Fenton reaction plus 2–8 µM rFA1654. Lane 7 (a–c) indicates that as little as 2 µM of rFA1654 can extinguish the effect of the Fenton reaction, resulting in the protection of DNA.

The ability of the FA1654 protein to protect DNA from oxidative stress‐induced damage was analyzed using a DNA protection assay in the presence of H_2_O_2_ and iron (Figure 3). Incubation of DNA in the presence of H_2_O_2_ showed minimal degradation (lane 3). This is in contrast to the complete degradation of the DNA in the presence of Fe_2_SO_4_ and H_2_O_2_ (lane 4). The DNA damage was significantly reduced when it was incubated with FA1654 (lanes 7–9) prior to the addition of H_2_O_2_ and Fe_2_SO_2_ (Figure 3). In the presence of a recombinant collagenase thioredoxin fusion protein (negative control), there was no observed protection against oxidative stress‐induced DNA damage (Figure 3, lane 5; Figure S1). Taken together, this may indicate the ability of the FA1654 protein to protect DNA from the Fenton reaction‐mediated damage. Moreover, under our experimental conditions, this may indicate that the thioredoxin fusion tag did not protect against the Fenton reaction‐mediated observed damage.

### 3.4 Inability of the rFA1654 protein to form DNA–DPS complexes

Although there were no predicted DNA binding domains in FA1654, its ability to bind/interact DNA was analyzed in the presence of H_2_O_2_ and iron. Similar to the control, incubation of the FA1654 protein with DNA resulted in no change in the migration pattern of the DNA through the agarose gel following electrophoresis (Figure 3). There was no observable change in the DNA migration pattern in the presence of iron and H_2_O_2_.

### 3.5 rFA1654 binds iron

**FIGURE 4:**
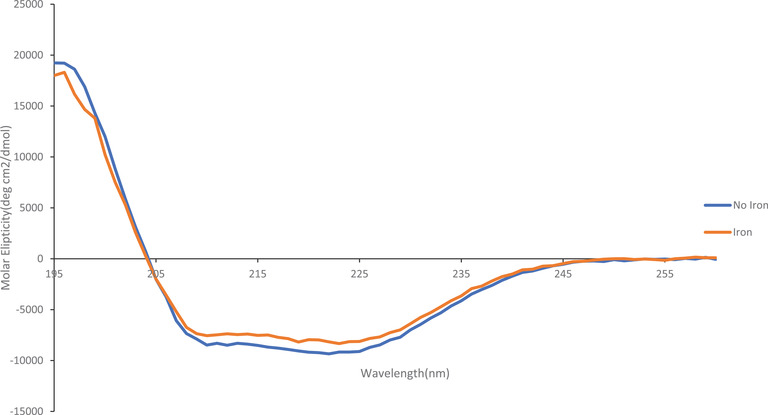
Circular dichroism (CD) analysis of rFA1654. CD spectra of rFA1654 and rFA1654 plus 50 µM Fe at 222 nm. Shift in the molar ellipticity indicates the binding of iron to rFA1654.

**FIGURE 5:**
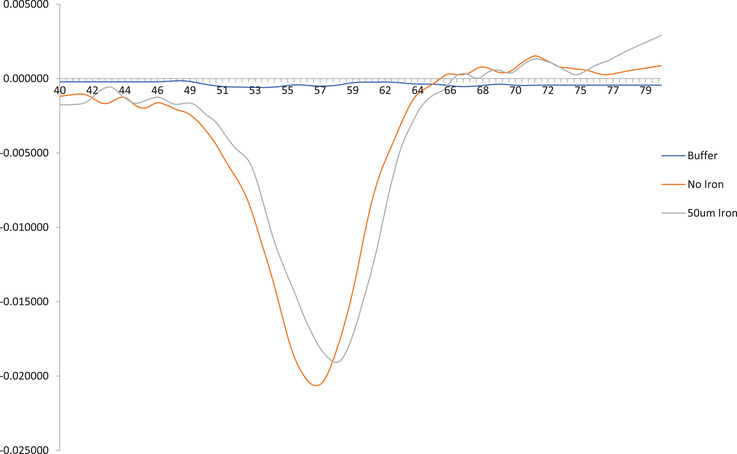
Differential scanning fluorimetry (DSF) analysis of rFA1654. DSF spectra of rFA1654 and rFA1654 plus 50 µM Fe. Thermal unfolding increased by ∼1.5°C, indicating the binding of Fe to rFA1654

**FIGURE 6:**
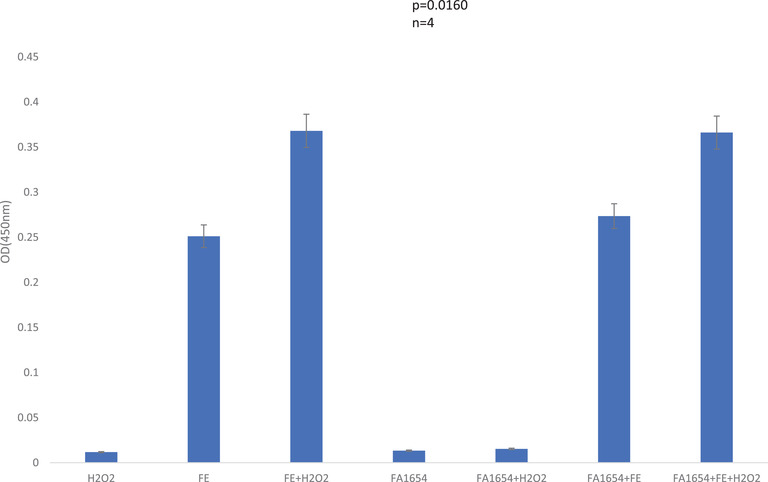
The ability of rFA1654 to reduce H_2_O_2_ was assessed using a 3,5,3′5′‐tetramethylbenzidine (TMB) assay. TMB when oxidized via cation radicals exhibits a colorimetric change from yellow to blue and can be detected at 450 nm. TMB was incubated in the presence of the Fenton reaction (Fe^2+^ and H_2_O_2_), TMB and H_2_O_2_ alone and TMB plus H_2_O_2_ plus rFA1654. The data obtained indicates that rFA1654 is unable to reduce H_2_O_2_.

The FA1654 protein is predicted to be an iron binding protein. To determine the ability of FA1654 to bind iron, we performed CD and differential scanning fluorimetry (DSF) in the absence or presence of iron. The far‐UV CD spectra exhibited two negative signals at 222 and 208 nm (Figure 4), which were consistent with significant helical structures in the protein. Deconvolution of the CD signals indicated the presence of 32% of α‐helicity in the absence of iron. Upon the addition of 50 µM Fe, the magnitude of the CD signal at 222 nm decreased by approximately 12.5%. This change suggested a small decrease in α‐helicity, which may be the result of binding of Fe to the protein (Figure 5). To corroborate this data, DSF was used to assess the effect of iron on the thermal stability of the protein. The thermal unfolding of rFA1654 was monitored by measuring the changes in intrinsic fluorescence at 330 and 350 nm (Figure 5). The DSF curve of rFA1654, which contains three Trp residues, displayed one unfolding transition that was centered at 54°C (this was considered its thermal unfolding temperature, *T*_m_). Addition of 50 µM Fe resulted in an increase in FA1654 *T*_m_ by approximately 1.5°C, suggesting thermostabilization of the native protein due to specific interactions with iron (Waldron & Murphy, 2003). Taken together, the results from CD and DSF analysis may provide evidence of the specific binding of iron to the native form of FA1654 (Figure 6).

### 3.6 H_2_O_2_ detection

A TMB assay was used to determine any intrinsic ability of the FA1654 protein to reduce H_2_O_2_ (Figure 6). The assay is based on the colorimetric change of the TMB reagent from yellow to blue when exposed to cation radicals such as OH•. Absorption spectroscopy measurement at 450 nm of TMB incubated in the presence of H_2_O_2_ and the FA1654 protein was 0.015 with no observed colorimetric change. This is in contrast to the control reaction of TMB incubated with Fe and H_2_O_2_ which resulted in an observed colorimetric change of yellow to blue with an OD_450 nm_ of 0.368. This data taken together may indicate that FA1654 may not have the intrinsic ability to reduce H_2_O_2_.

## 4 DISCUSSION

Oxidative stress can play a role in the progression of periodontal disease, which is known to affect more that 65 million adults in the United States (Aja, Mishra, et al., 2021; Eke et al., 2015; Mehrotra & Singh, 2019). The impact of the microbial‐induced host response involving the generation of ROS, such as H_2_O_2_ and hydroxyl radicals, is important to disease development/progression (Sczepanik et al., 2020). Recently described as part of the “Inflammation‐Mediated Polymicrobial‐Emergence and Dysbiotic‐Exacerbation” Model proposes there is a central role for inflammation and its ability to modulate the polymicrobial community along a health to periodontitis continuum (Van Dyke et al., 2020). As part of this process that can trigger a dysbiotic state to initiate periodontitis, there is inflammation‐mediated microbial succession that drives the temporal and spatial emergence of disease‐associated species in the periodontal pocket. With disease progression, the transitional dysbiotic microbiota is further altered by an increase in abundance of predominantly disease‐associated species particularly at the base of the periodontal pocket resulting in an opportunistic polymicrobial synergistic infection and correlated with an excessive inflammatory response with the accompanying release of ROS, such as H_2_O_2_ and superoxide radicals from neutrophils and concomitant tissue destruction (Armstrong et al., 2018; Mishra et al., 2020; Sczepanik et al., 2020). There is evidence that *F. alocis* can successfully survive and co‐occur with other previously recognized periodontal pathogens in the oxidative stress rich environment of the periodontal pocket.

This study has provided insights into the role of a *F. alocis* hypothetical protein with DPS‐like characteristics, involved in oxidative stress resistance via an ability to protect against Fenton reaction‐mediated oxidative stress‐induced DNA damage. We have shown that the FA1654 protein share identity to several DPS proteins, including the DPS from *Fusobacterium* spp., *Clostridiales bacterium*, and *T. pedis*. The FA1654 protein is predicted to have an iron binding domain and thus is considered to be a putative DPS protein. DPS proteins are associated with the protection of DNA from Fenton reaction‐mediated oxidative stress (Antipov et al., 2017; Bellapadrona et al., 2010) via DNA binding and/or iron binding (Antipov et al., 2017; de Alcântara et al., 2020; Tseng et al., 2019). Our studies have indicated that FA1654 can bind iron in vitro. Iron has been demonstrated to be a critical element in the bacterial growth, survival, and pathogenesis (Andrews et al., 2003; Frawley & Fang, 2014). However, under conditions such as oxidative stress, iron leads to damage due to its role in the Fenton reaction and the resulting generation of ROS. There are three major cellular protective systems against oxidative stress in bacteria. These include the DNA repair, chaperone/protease, and antioxidant systems (McKenzie et al., 2012). In one of the major antioxidant defenses, iron is carefully sequestered within proteins and is restricted from reacting with ROS (Gutteridge & Halliwell, 2018). Driven by the ability of ROS to bind and oxidize ferrous iron, oxidative stress is a feature of iron metabolism. When H_2_O_2_ reacts with DNA‐bound iron, it generates mutagenic and even lethal DNA lesions. The rFA1654 protein in this study protected DNA from Fenton reaction‐mediated damage. The damage protection in the presence of H_2_O_2_ did not likely occur via an ability of the FA1654 protein to bind DNA. Consistent with the absence of any predicted DNA binding domains in FA1654, an ability to bind DNA was not observed.

**FIGURE 7:**
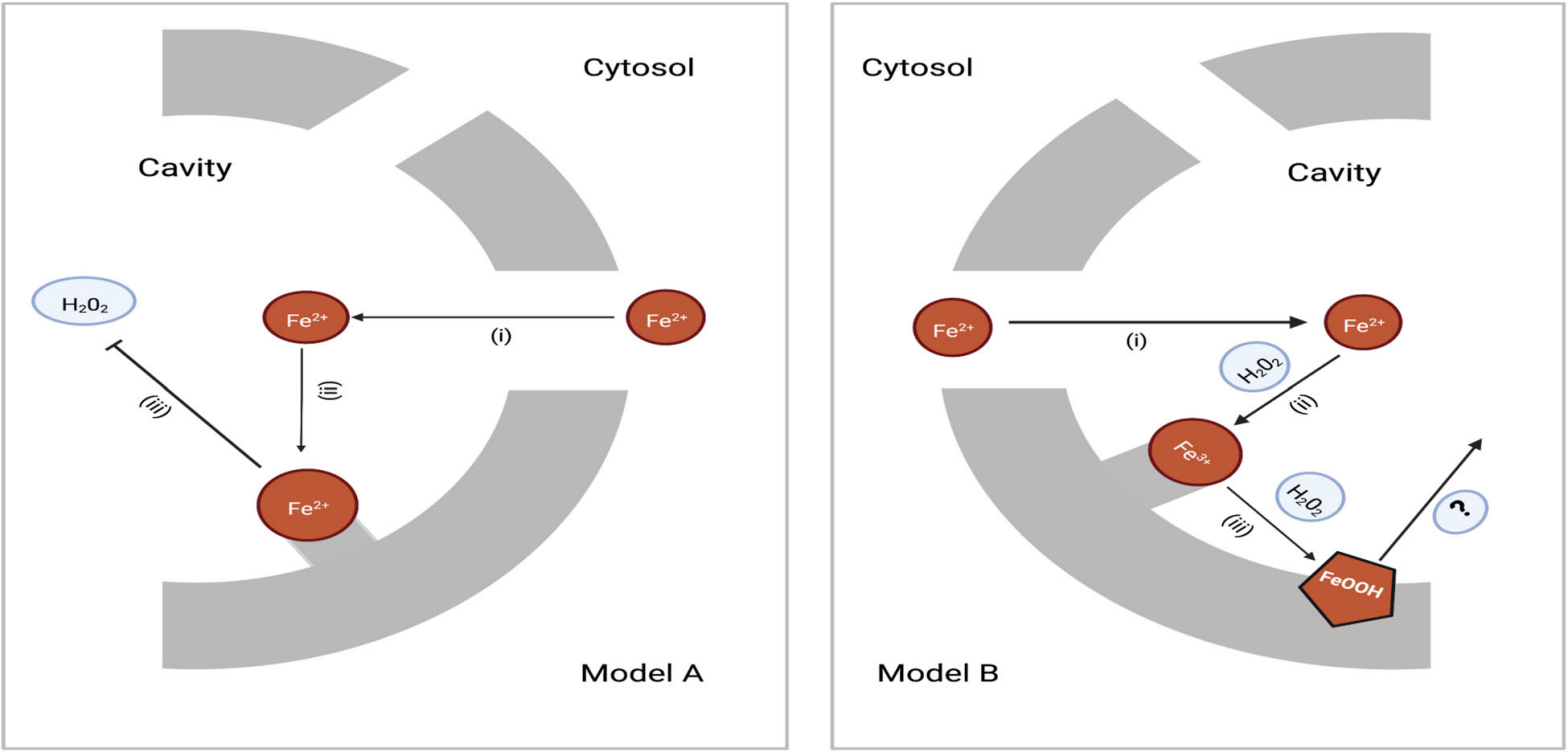
Proposed mechanism(s) of action for FA1654. FA1654 binds iron via its di‐iron binding site. (1) This iron may be bound in the 2+ state and therefore may unable to react with H_2_O_2_, minimizing the generation of Fenton‐mediated reactive oxygen species (ROS) (Model A). (2) It is also possible that the FA1654 protein may utilize available H_2_O_2_ as an oxidant, oxidizing iron to produce an iron oxide hydroxide (Model B). The mechanism of iron homeostasis in *F. alocis* is currently not fully understood, it is yet to be determined if bound iron can be recycled and the mechanism by which it occurs. Experiments to determine the amino acid residues involved in iron binding and the kinetics of iron bound to the protein are required to fully elucidate the proposed mechanism.

The ability of the FA1654 to bind iron, similar to other DPS proteins described in *E. coli*, *Listeria innocua*, and *Campylobacter jejuni* (Almiron et al., 1992; Huergo et al., 2013; Ishikawa et al., 2003; Yang, Chiancone, et al., 2000), could likely indicate a sequestration function. Thus, a possible mechanism of action of FA1654 is that the binding of iron to a putative active site which could render it unavailable to interact with H_2_O_2_ and possibly preventing the generation of free radicals such as OH•. It is also likely that the protein may utilize H_2_O_2_ as an oxidant in the multistep oxidation of iron from its 2^+^ to 3^+^ state (de Alcântara et al., 2020) to generate the formation of Iron(III) oxide–hydroxide FeO(OH). This nonreactive form, as the end product of iron oxidation, could be utilized by *F. alocis* proteins to sequester free iron (Haikarainen & Papageorgiou, 2010; Yang et al, 1998; Yang, Chiancone, et al., 2000; Yang, Le Brun, et al., 2000) (Figure 7). Taken together, the sequestering of iron and the formation of FeO(OH) may limit the interation between iron and H_2_O_2_, reducing the generation of free radicals. FA1654 was also unable to detoxify H_2_O_2_, suggesting that it has no intrinsic peroxidase activity which could further support its possible role in iron oxidation (Yang, Le Brun, et al., 2000). It is unclear if FA1654 may have other functions or can modulate the iron pool within the cell. Because oxidative stress‐induced DNA damage is directly proportional to the amount of loose iron in the cell, an ability to scavenge H_2_O_2_ is important for growth and survival under those environmental conditions. Moreover, oxidants can damage iron enzymes, resulting in the released and severalfold increase in free available iron (Keyer & Imlay, 1996). It is likely that FA1654 is part of a complex system in *F. alocis* given it relative resistance to oxidative stress (Aja, Mangar, et al., 2021). Although H_2_O_2_ scavenging enzymes, in most anaerobic organisms, include catalases, peroxidases bacterioferritin comigratory protein, ruberythrin, and alkyl hydroperoxide reductase enzyme system (AhpC/AhpF) (Aja, Mangar, et al., 2021; Mishra et al., 2020; Mishra & Imlay, 2012), several are missing from the genome of *F. alocis* (Mishra et al., 2020). The *F. alocis* SOR (FA796) protein is a key enzymatic scavenger of superoxide radicals and can protect the bacterium from oxidative stress conditions (Mishra et al., 2020). The multifunctional *F. alocis* FA519 protein, which may represent a novel class of thioredoxin family proteins, can protect DNA from Fenton reaction‐mediated damage with intrinsic peroxidase and disulfide oxidoreductase activities (Aja, Mishra, et al., 2021). It is worth noting that the genome of *F. alocis* carries the genes for iron–sulfur cluster assembly, ferrous iron transport, FTR1 family, and DprA proteins all of which are currently uncharacterized. Additionally, *F. alocis* encodes for glutathione peroxidase, alkyl hydroperoxide reductase subunit AhpC, and thioredoxin‐disulfide reductase (Aja, Mangar, et al., 2021) genes which may play a role in H_2_O_2_ scavenging. Together, in *F. alocis*, these systems along with FA1654 may be part of a complex network with unknown regulatory components. Although we have made several unsuccessful attempts to inactivate the *FA1654 gene* in *F. alocis*, studies to further validate its role in modulating the response of the bacterium to the oxidative stress environment of the periodontal pocket are ongoing in the laboratory.

Collectively, the data obtained from this study suggests that *F. alocis* FA1654 may be a member of the ferritin superfamily and a possible DPS. It protects DNA from Fenton reaction‐mediated damage and has the intrinsic ability to bind iron. The ability for FA1654 to modulate important virulence attributes in *F. alocis* is still unclear. What role does FA1654 play in iron homeostasis and how is it regulated in *F. alocis*? Can sequestered iron be released in times of iron deficiency (de Alcântara et al., 2020)? The specific function and relative significance of FA1654 in *F. alocis* is under further investigation in the laboratory.

## AUTHOR CONTRIBUTIONS

Malissa Mangar, Arunima Mishra, Zhengrong Yang, Champion Deivanayagam, and Hansel M. Fletcher conceived and designed the experiments. Malissa Mangar and Zhengrong Yang conducted experiments, collected, and analyzed the data. Malissa Mangar wrote the original draft. Hansel Fletcher validated the data and modified the manuscript. All authors have read and approved the final manuscript.

## CONFLICT OF INTEREST

The authors declare no conflict of interest.

### PEER REVIEW

The peer review history for this article is available at https://publons.com/publon/10.1111/omi.12398.

## Supporting information

Supporting Information

## Data Availability

The data generated or analyzed during this study is included in this article.
