# Supplementary material for: Characterization of FA1654: A putative DPS protein in Filifactor alocis
Source: Mol Oral Microbiol. 2022 Dec 19;38(1):23–33. doi: 10.1111/omi.12398 (PMC9905271; doi:10.1111/omi.12398)
Supplement: Supplementary file 1 — Supporting Information [file OMI-38-23-s001.pdf]

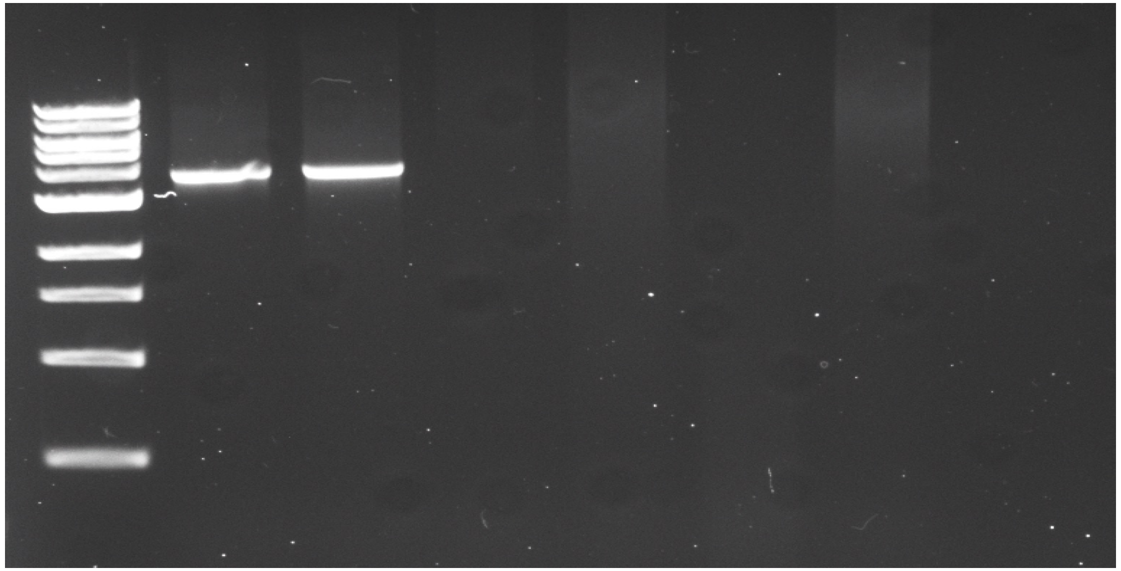

	RNA Pol B					
	2	3	4	5	6	7
DNA	✓	✓	✓	✓	✓	✓
H_2O_2		✓	✓	✓	✓	✓
$FeSO_4$			✓	✓	✓	✓
FA1750A				✓		
FA1006					✓	
FA1336						✓

Supplemental Fig 1. DNA protection assay using collagenase- thioredoxin fusion proteins as a control. Data indicates the proteins do not have the ability to protect DNA and its thioredoxin tag is inactive.
